# Simplifications and approximations in a single-gene circuit modeling

**DOI:** 10.1038/s41598-024-63265-8

**Published:** 2024-05-31

**Authors:** Alejandro Barton, Pablo Sesin, Luis Diambra

**Affiliations:** 1 Centro Regional de Estudios Genómicos, Universidad Nacional de La Plata, La Plata, Argentina; 2 Consejo Nacional de Investigaciones Científicas y Técnicas, Buenos Aires, Argentina; 3 Departamento de Física Teórica, GAIDI, Comisión Nacional de Energía Atómica, 1429 Buenos Aires, Argentina

**Keywords:** Systems biology, Biochemical networks, Control theory, Genetic circuit engineering, Nonlinear dynamics, Oscillators, Regulatory networks, Biological physics, Statistical physics, thermodynamics and nonlinear dynamics

## Abstract

The absence of detailed knowledge about regulatory interactions makes the use of phenomenological assumptions mandatory in cell biology modeling. Furthermore, the challenges associated with the analysis of these models compel the implementation of mathematical approximations. However, the constraints these methods introduce to biological interpretation are sometimes neglected. Consequently, understanding these restrictions is a very important task for systems biology modeling. In this article, we examine the impact of such simplifications, taking the case of a single-gene autoinhibitory circuit; however, our conclusions are not limited solely to this instance. We demonstrate that models grounded in the same biological assumptions but described at varying levels of detail can lead to different outcomes, that is, different and contradictory phenotypes or behaviors. Indeed, incorporating specific molecular processes like translation and elongation into the model can introduce instabilities and oscillations not seen when these processes are assumed to be instantaneous. Furthermore, incorporating a detailed description of promoter dynamics, usually described by a phenomenological regulatory function, can lead to instability, depending on the cooperative binding mechanism that is acting. Consequently, although the use of a regulating function facilitates model analysis, it may mask relevant aspects of the system’s behavior. In particular, we observe that the two cooperative binding mechanisms, both compatible with the same sigmoidal function, can lead to different phenotypes, such as transcriptional oscillations with different oscillation frequencies.

## Introduction

In recent times, mathematical modeling has emerged as a pivotal instrument in contemporary Biology. In numerous instances, a quantitative approach in Molecular Systems Biology is mandatory to understand the mechanisms that drive many of the observed phenomena^1,2^. In general, systems are constituted of a substantial number of heterogeneous elements, and certain simplifications are needed to obtain a more manageable model. Here, simplification refers to the elimination of intricacies and details from the model that are perceived to have an insignificant impact on the replication of a specific aspect of interest in the system under study. Despite these simplifications, some models remain analytically intractable due to the sheer number of variables, parameters, and inherent non-linearities that increase the complexity, requiring sophisticated mathematical techniques at times. Further, incomplete knowledge of the underlying interaction mechanisms often requires the use of phenomenological assumptions, like regulatory functions. Nevertheless, models often require mathematical approximations, such as the time scale separation methods, as a means of achieving feasibility. If pertinent assumptions are made, it is expected that the abridged version of a model will furnish consistent outcomes with its detailed counterpart; and modifications to model components, such as a change in parameters, should mimic modifications to the real system. However, the simplifications and approximations discussed above impose limits on the biological interpretation of processes and parameters used in the model. In this paper, we examine a single-gene oscillator to assess the implications, limitations, and potential misapplications of common assumptions and approximations in molecular systems biology modeling within a deterministic framework. Modeling single-gene oscillators can be more than a propaedeutical way to introduce systems biology modeling; in fact, it is the core circuit that drives somitogenesis during vertebrate embryogenesis^3^. This genetic timekeeper is believed to be driven by the self-regulation of her/hes genes, which contain multiple regulatory binding sites for inhibition^4^. In the last decade, this topic has attracted the attention of both theoretical and experimental researchers, and several studies have focused on segmentation clock modeling^5–9^. Thus, models including mechanistic details, as proposed here, could be a suitable platform for further studies of vertebrate segmentation clocks.

In exploring genetic oscillator modeling, it is essential to consider fundamental mathematical principles such as the Bendixson–Dulac theorem^10^. This theorem provides the necessary conditions for excluding oscillatory solutions in systems described by two nonlinear ordinary differential equations. Specifically, in the context of single-gene models, this theorem grants that oscillations are impossible when considering only two chemical species, independent of the non-linearity present in the model. Thus, it highlights the necessity of extending models to include detailed mechanisms that can exhibit dynamic behaviors not accounted for by simpler models. In this sense, Goodwin proposed a model with three variables (processes) and high non-linearity mainly embedded in a Hill function^11^. This model can exhibit oscillatory behavior. However, its soundness as a gene oscillator model has been challenged on account of the excessively high value that the Hill exponent must take to display sustainable oscillations. The matter has unleashed some controversy about the number of processes that must be considered in a reliable oscillatory model to observe oscillatory behavior. Several papers have introduced some other mechanisms to reach such oscillations. These mechanisms can consider an increasing number of components^12–14^ or include improvements in the descriptive level of the systems. In this last sense, it has been proven that taking into account ingredients such as delayed variables^15^, involved in synthesis, transport or intrinsic stochastic noise^16^, cis-regulatory sites for TF genes^16–18^ or protein–protein interaction can lead these auto-inhibitory circuits to instability^19^. Further, it has been proposed the use of cascades of post-translational covalent modifications, instead of a transcriptional regulatory function, as a non-linearity source^12^. However, more recently it has been demonstrated that oscillations may arise due to a global physiological response, rather than a specific molecular mechanism^20^.

The implementation of time-lagged variables has emerged as an alternative solution to reliable transcriptional oscillator models^15,21,22^. The inclusion of delay variables is a pivotal factor in introducing non-linearity into models, highlighting the significance of time delays in the modeling of transcriptional oscillators. Often, the introduction of this kind of variable is made as a discrete single delay to substitute one or more processes (such as transcript elongation, translation, or translocation) with an equivalent characteristic time. However, this substitution in not always justified and a distributed delay approximation should be more adequate to represent such processes. Mathematically, a discrete delay represents an infinite number of processes and carries implicitly a high non-linearity. Thus, this approximation can lead to inaccurate results when interpreting the parameters of the modeling.

Here, besides considering distributed delays for synthesis and degradation processes, we also introduce a detailed description for the *cis* regulatory system (CRS) in a single-gene oscillatory circuit. Our findings reveal that the phenomenological simplification of the regulatory function operating in the system can obscure a range of possible scenarios. The outline of the paper is as follows: in “Modeling transcriptional oscillations with instantaneous processes”, we introduce a series of models for an autoinhibitory circuit of a single gene, inspired by the Lewis segmentation clock model^15^. These three models capture the dynamics of mRNA and protein synthesis/degradation with an increasing level of description. In these cases, the autoinhibitory mechanism is modeled by a phenomenological Hill function. We demonstrate that different levels of detail can predict divergent phenotype behaviors for the regulatory circuit analyzed, ranging from a stable node and stable spiral to sustained oscillations. In “Single-gene oscillator models with explicit CRS dynamics”, we disaggregate the binding/unbinding processes associated with CRS dynamics and replace the regulatory function with three new differential equations. From this model, we explore different delay approximations by implementing various delay kernels that weigh the effects of past concentrations on the current state. We find that all these kernels lead to the same fixed point, but its stability, and the potential for oscillations, depend on the order of the delay kernel used. Interestingly, the detailed description of the CRS reveals differences in amplitude and frequency between phenotype behaviors resulting from two different cooperative binding mechanisms proposed in^23^, even when these mechanisms are associated with the same regulatory function. These differences are overlooked when using the instantaneous approach for modeling transcriptional regulation. The significance of this finding is discussed in the last section.

## Modeling transcriptional oscillations with instantaneous processes

**Figure 1:**
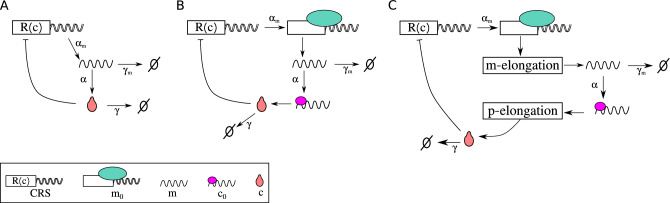
The sketches of autorepressive single-gene circuit from the perspective of three description levels. In model I the transcription and translation are described as instantaneous processes that occur at an average rate of $\alpha _m$ and $\alpha$, respectively (**A**). Model II includes the open state of DNA ($m_0$), and the translation initiation complex ($c_0$) (**B**). Model III considers the elongation processes associated with mRNA and proteins represented by boxes (**C**). Note that elongation processes can be mathematically described by a dedicated ODE for each step (Eq. 3) or by a set of differential equations with delays (Eq. 6). In three cases transcript and proteins are degraded following first-order reactions with rates $\gamma _m$ and $\gamma$, respectively. In all three cases, the transcription is regulated following a repressive Hill function.

We will consider a generic single-gene model that describes the synthesis and degradation of its associated transcript and protein. This hypothetical gene encodes a transcription factor (TF) that regulates negatively its own transcript synthesis, thus forming a feedback loop. The ODEs that govern this circuit can be written as:

1 $$\begin{aligned} \dot{m}= & {} \alpha _m R\left( c\right) - \gamma _m m, \nonumber \\ \dot{c}= & {} \alpha \ m - \gamma \ c, \end{aligned}$$

where *m* and *c* represent the concentration of messengers and TF, respectively. The complex processes of transcription and translation are described as instantaneous processes that occur at an average rate of $\alpha _m$ and $\alpha$, respectively. Degradation processes are considered linear with an average rate of $\gamma _m$ for transcripts and $\gamma$ for proteins. Figure 1A illustrates this simple model. *R* is the regulatory function, which is monotonically decreasing in the case of auto-inhibitory circuits. In Eq. (1), hereafter model I, the regulatory function *R* can be understood as the result of many molecular processes. For the sake of model simplicity, these processes are not described explicitly, but through a phenomenological expression. Many times, the regulatory function used in biological modeling corresponds to a sigmoidal function, often the Hill function $R\left( c\right) =1/(1+ (c/K_d)^{n_H})$ where $n_H$ is the Hill exponent, and $K_d$ is the apparent dissociation constant. This regulatory function represents the action of transcription factors interacting with the CRS of the regulated gene^23^, and will be discussed further.

Now, we will introduce two models with higher descriptive levels for the transcription and translation processes, to show how a common simplification of considering a complex process as instantaneous can lead to different scenarios. In the first case, we split the transcription process of the circuit above into two steps, by considering the formation of protein–DNA complexes that repress transcription, and the elongation process separately. Performing a similar split for protein synthesis, we can write the model II as

2 $$\begin{aligned} \dot{m}_0= & {} \alpha _m \ R(c) - \beta _1 \ m_0 \nonumber \\ \dot{m}= & {} \beta _1 m_0 - \gamma _m \ m \nonumber \\ \dot{c}_0= & {} \alpha \ m - \beta _2 \ c_0 \nonumber \\ \dot{c}= & {} \beta _2 \ c_0 - \gamma \ c, \end{aligned}$$

where $m_0$ denotes the open state of DNA, $c_0$ denotes the translation initiation complex. $\beta _1$ and $\beta _2$ are the average elongation rates for transcription and translation, respectively. *m* and *c* represent the free transcripts and peptides. A sketch of model II is illustrated in Fig. 1B.

Alternatively, we can increase the description level of the model by adding a step-by-step elongation process associated with transcripts and proteins. In this case, we can rewrite the two equations in Eq. (1) in the form

3 $$\begin{aligned} \dot{m}_0= & {} \alpha _m \ R(c) - r_1 \ m_0 \nonumber \\ \dot{m}_i= & {} r_1 \ m_{i-1} - r_1 \ m_i \ \ \ \text{with} \ \ i=1,\ldots ,N \nonumber \\ \dot{m}= & {} r_1 \ m_{N} - \gamma _m \ m \nonumber \\ \dot{c}_0= & {} \alpha \ m - r_2 \ c_0 \nonumber \\ \dot{c}_j= & {} r_2 \ c_{j-1} - r_2 \ c_j \ \ \ \text{with} \ \ j=1,\ldots ,M \nonumber \\ \dot{c}= & {} r_2 \ c_{M} - \gamma \ c, \end{aligned}$$

where $r_1$ and $r_2$ are the step elongation rates for transcription and translation, respectively. ${m}_i$ (${c}_i$) represents transcripts (peptides) with *i* (*j*) nucleotides (amino acids). This model is schematized in Fig. 1C. At this point, we introduce the linear chain trick^24,25^, for one single transcript elongation step *i* we have

$$\begin{aligned} \dot{m}_i = r_1 \ m_{i-1} - r_1 \ m_i \longrightarrow m_i(t)=\int _{-\infty }^t r_1 e^{-r_1(t-s)} m_{i-1}(s) ds \end{aligned}$$

and two consecutive elongation steps

$$\begin{aligned} \dot{m}_i = r_1 \ m_{i-1} - r_1 \ m_i \\ \dot{m}_{i+1} = r_1 \ m_{i} - r_1 \ m_{i+1} \end{aligned}$$

can be written in terms of the previous one as

$$\begin{aligned} m_{i+1}(t)=\int _{-\infty }^t r_1^2 (t-s) e^{-r_1(t-s)} m_{i-1}(s) ds. \end{aligned}$$

Thus, by using the linear chain trick both for $m_{N}$ and $p_{M}$ and by changing variable $(t-s) \longrightarrow \tau$ we obtain

$$\begin{aligned} m_{N}\left( t\right) =\int _0^{\infty } K_{r_1}^{N} (\tau ) \ m_0\left( t-\tau \right) d\tau \\ c_{M}\left( t\right) =\int _0^{\infty } K_{r_2}^{M} (\tau ) \ c_0\left( t-\tau \right) d\tau , \end{aligned}$$

where

4 $$\begin{aligned} K_r^n (\tau )= \frac{r^n \tau ^{n} e^{-r\tau }}{n!}, \end{aligned}$$

is the Gamma distribution delay kernel of order *n*. A delay kernel is a weighting function that indicates how much emphasis should be given to the concentrations at earlier times to determine the present effect. Thus, Eqs. (3) can be reduced to

5 $$\begin{aligned} \dot{m}_0= & {} \alpha _m \ R(c) - r_1 \ m_0 \nonumber \\ \dot{m}= & {} r_1 \ m_{N} - \gamma _m \ m \nonumber \\ \dot{c}_0= & {} \alpha \ m - r_2 \ c_0 \nonumber \\ \dot{c}= & {} r_2 \ c_{M} - \gamma \ c, \end{aligned}$$

plus the integrals for $m_{N}$ and $c_{M}$. By replacing the integrals for $m_{N}$ and $c_{M}$ into Eq. (5) we would obtain a set of distributed delay differential equations. The discrete delay can be recovered as a limit of the Gamma distributed delay if the mean delay remains *n*/*r* but the variance goes to zero when $n\longrightarrow \infty$. Thus, if sequences are long enough, one can approximate the distributed kernels above by discrete delays with the mean delay $\tau _N= N/r_1$ and $\tau _M= M/r_2$. Thus, the model III is given by

6 $$\begin{aligned} \dot{m}_0= & {} \alpha _m \ R(c) - r_1 \ m_0 \nonumber \\ \dot{m}= & {} r_1 m_0 \left( t-\tau _N \right) - \gamma _m \ m \nonumber \\ \dot{c}_0= & {} \alpha \ m - r_2 \ c_0 \nonumber \\ \dot{c}= & {} r_2 \ c_0\left( t-\tau _M \right) - \gamma \ c, \end{aligned}$$

**Figure 2:**
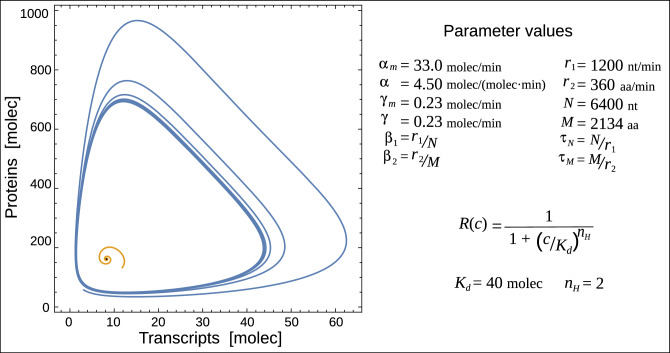
The dynamics of a transcriptional oscillator from the perspective of three description levels. Trajectories in the phase plane for three models. The parameter values used in these simulations are the same values that were used in^15^ and are listed in the right panel. Model I has a stable fixed point (black dot), model II exhibits stable spiral behavior (yellow line), and model III exhibits sustainable oscillations (blue line). The initial conditions used for models I and II are $m_0(0)= 1.0$ and $m(0)=c_0(0)=c(0)=0$. The initial conditions for delay model III are $m_0= 1.0$ for $-\infty < t \le 0$, while *m*, $c_0$ and *c* are zero in such interval. The temporal range of the plot is from $50> t > 500$. The effective elongation rates in model II have been written in terms of the single elongation step, as $\beta _1=r_1/N$ and $\beta _2=r_2/M$, where *N* and *M* denote the transcript and protein lengths.

Models I, II, and III represent the same system but at different description levels. All models share the same fixed point, however, the stability of this point depends on the description level of the model. Figure 2 depicts the behavior of models obtained by numerical integration using the same parameter values of the Lewis segmentation clock^15^. Whereas the last model exhibits sustainable oscillations (blue line), model II exhibits stable spiral behavior (yellow line), and model I has a stable fixed point (black dot).

The model II can display sustainable oscillations for higher values of the Hill exponent ($n_H >4$); consequently, if one intends to obtain dynamics that emulate experimentally observed oscillations with this model, can lead to an overestimation of the Hill exponent. This exercise provides evidence that considering processes that involve several steps as a single instantaneous step with an effective parameter can lead to wrong conclusions. Hereafter, we will refer to this simplification as the instantaneous simplification. Note that one can recover model I by applying the quasi-steady-state approximation on any of the models II and also from Eq. (3). The instantaneous simplification is present in almost all terms in the model I. Of course, simple models are preferred, but the simplicity of the model must be balanced against its predictive power, and minor aspects that do not affect the predictions can be left out.

The main point of this paper is focused on the term representing the regulation of transcript synthesis, *R*(*c*). In the next sections, we will see that instantaneous simplifications hide alternative phenotypes linked to two cooperative binding mechanisms. Some years ago, the recruitment and stabilization binding mechanisms were reported to be associated with the same regulatory function and have associated different levels of noise^23^. We will show that the stability of the fix-point in single-gene systems depends on which of these mechanisms is acting and this revelation is exposed only for models with a more detailed description of CRS dynamics.

## Single-gene oscillator models with explicit CRS dynamics

In the previous section, we presented models where the regulation of gene expression is represented by only one step. However, we can break down the complex processes involved in transcriptional regulation, usually represented by a phenomenological regulatory function. Let’s consider a CRS with three regulatory binding sites. TFs can bind or unbind to regulatory sites following the law of mass action for elementary reactions. Further, the transcription process occurs only when all regulatory sites are vacant, leading to the formation of a negative feedback loop. Mathematically, this model can be written as

7 $$\begin{aligned} \dot{a_0}= & {} -k_{01} a_0 c + k_{10} a_1 \nonumber \\ \dot{a_1}= & {} -k_{12} a_1 c + k_{21} a_2 + k_{01} a_0 c - k_{10} a_1 \nonumber \\ \dot{a_2}= & {} -k_{23} a_2 c + k_{32} a_3 +k_{12} a_1 c - k_{21} a_2\nonumber \\ \dot{a_3}= & {} k_{23} a_2 c - k_{32} a_3 \nonumber \\ \dot{ c}= & {} \alpha a_0 - \gamma c + k_{10} a_1 + k_{21} a_2 + k_{32} a_3 - c (k_{01} a_0 + k_{12} a_1 + k_{23} a_2), \end{aligned}$$

where *c* is the concentration of the TF, $a_i$ is the fraction of genes with *i* bound TFs, and $k_{i,i+1}$ are the kinetic rates for TF binding to DNA, while $k_{i+1,i}$ are the kinetic rates for TF unbinding. We can note that there is a conserved quantity, $1 = a_0 + a_1 + a_2 + a_3$. We will assume that the amount of *c* recruited(released) by binding(unbinding) to(from) regulatory sites is negligible, and we approximate the last equation in (7) obtaining the model IV:

8 $$\begin{aligned} \dot{a_0}= & {} - k_{01} a_0 c + k_{10} a_1 \nonumber \\ \dot{a_1}= & {} - k_{12} a_1 c + k_{21} a_2 + k_{01} a_0 c - k_{10} a_1 \nonumber \\ \dot{a_2}= & {} - k_{23} a_2 c + k_{32} (1 - a_0 - a_1 - a_2) + k_{12} a_1 c - k_{21} a_2 \nonumber \\ \dot{c}= & {} \alpha a_0 - \gamma c. \end{aligned}$$

A representation of this model is depicted in Fig. 4A.

Before considering the stability of this model, let us regard the cooperative interactions between TFs in detail following^23^ and assume for the sake of simplicity that all binding sites are identical. In the case of cooperative binding, the kinetic rates $k_{i,j}$ are not independent because the interactions between TFs alter the new binding or unbinding processes^26^. The thermodynamic relationship and the system’s kinetics allow us to write the kinetic rates $k_{i,j}$ in terms of only three parameters^27^: the binding rate *p*, the unbinding rate *q*, and the cooperativity intensity $\epsilon =e^{-\frac{\Delta G_\text{I}}{RT}}$, where $\Delta G_\text{I}$ is the free energy among TFs interaction, *R* is the gas constant, and *T* is the temperature. These relationships allow the identification of two cooperative binding mechanisms: the recruitment and stabilization mechanisms. The first mechanism corresponds to the case when the already bound TFs enhance the ability for new TF recruitment for DNA binding, increasing kinetic rates $k_{i,i+1}$. On the other hand, the stabilization mechanism acts when TF interaction diminishes the kinetic rates $k_{i+1,i}$. In this manner, following^23^, we can write:

9 $$\begin{aligned} {k_{i,i+1}}= & {} \epsilon ^{i} \left( 3-i\right) p \nonumber \\ {k_{i+1,i}}= & {} (i+1) q, \ \ \ \ \ i=0,1,2, \end{aligned}$$

for the first mechanism, while for the second mechanism we have

10 $$\begin{aligned} {k_{i,i+1}}= & {} \left( 3-i\right) p, \nonumber \\ {k_{i+1,i}}= & {} (i+1) q/ \epsilon ^{i}, \ \ \ \ \ i=0,1,2. \end{aligned}$$

**Figure 3:**
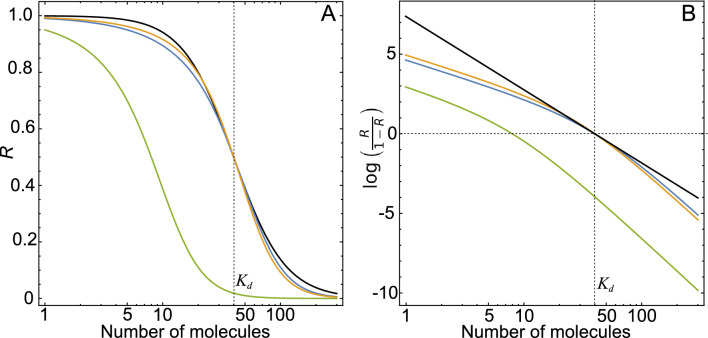
Transcriptional regulatory functions for a repressor. The Hill function used in Fig. 2 ($K_d=40$ and $n_H=2$, black curve). Three Adair regulatory functions with different parameter values: $q = 31.3$, $\epsilon = 5.5$ (blue curve), $q = 43$, $\epsilon = 8.5$ (yellow curve), and $q = 6.26$, $\epsilon = 5.5$ (green curve) and $p = 0.1$ for all cases. The associated phenomenological parameters are $K_d=40$ $n_H=1.96$, $K_d=40.1$ $n_H=2.21$ and $K_d=8$, $n_H=1.96$, respectively (**A**). The same regulatory functions, but in the Hill plot style, where the vertical axis is the transformed receptor occupancy, i.e., $Log[\frac{R}{1-R}]$. This plot better demonstrates the difference between Hill and Adair’s regulatory functions. While the slope of a Hill function is constant and equal to the Hill coefficient $n_H$, this slope varies in the case of the Adair function (**B**).

When the TF binding or unbinding to the regulatory sites is quick regarding the synthesis and degradation processes, one can use the quasi-steady-state (QSS) approximation and obtain an approximated model, as in the previous section. The quasi-steady-state solution can be obtained by replacing the left-hand side of the equations above with 0 and solving the resulting algebraic equations. After some algebraic steps, we obtain that the approximated model is given by

11 $$\begin{aligned} \dot{c} = \alpha R^{qss}(c) - \gamma c, \end{aligned}$$

where $R^{qss}(c)$ is the regulatory function obtained from the CRS dynamics in model IV. It is known as the Adair equation^28^ and takes the form of a sigmoidal function, $R^{qss}(c)=\left( 1+ c K_{1} + c^2 K_{1} K_{2} + c^3 K_{1} K_{2} K_{3} \right) ^{-1}$ where $K_1=k_{01}/k_{10}, \ K_2=k_{12}/k_{21}$ and $K_3=k_{23}/k_{32}$ are the equilibrium constants. Note that in QSS approximation, the regulatory function depends only on the kinetic parameters through the equilibrium constants $K_i$ but not on the kinetic rates $k_{i,j}$. In the limit of the high interaction energy between TF molecules, where $K_3>>K_2, \ K_1$, the regulatory function resembles the phenomenological Hill function used in the models of the previous section. For the sake of comparison, Fig. 3 illustrates both types of regulatory functions: the Hill function (black curves) and the Adair regulatory function for three sets of parameter values. Model IV differs from the model in Eq. (11) in that the transcriptional regulation process is not an instantaneous one, but both models have the same steady state, which is asymptotically stable in all cases (see the stability analysis of model IV in Appendix A of Supplementary Material).

In the next step, we build up model V by splitting the gene expression process into the transcription and translation steps as follows:

12 $$\begin{aligned} \dot{a_0}= & {} -k_{01} a_0 c + k_{10} a_1 \nonumber \\ \dot{a_1}= & {} -k_{12} a_1 c + k_{21} a_2 + k_{01} a_0 c - k_{10} a_1 \nonumber \\ \dot{a_2}= & {} -k_{23} a_2 c + k_{32} (1 - a_0 - a_1 - a_2) +k_{12} a_1 c - k_{21} a_2 \nonumber \\ \dot{m}= & {} \alpha _m a_0 - \gamma _m m \nonumber \\ \dot{c}= & {} \alpha \ m - \gamma \ c. \end{aligned}$$

**Figure 4:**
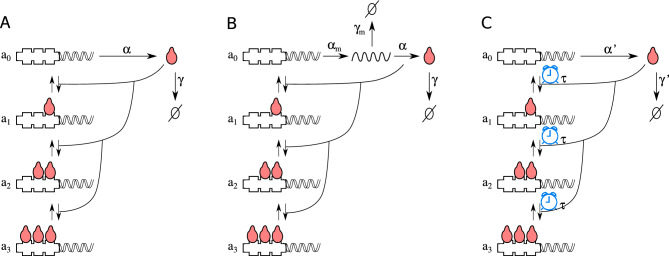
Three autorepressive single-gene circuit models with explicit CRS dynamics. Model IV considers a CRS with three identical regulatory sites for FT that inhibit gene expression. The gene can only be expressed when the CRS has no bound TF. The TF expression occurs at an average rate of $\alpha$, and it is degraded at rate $\gamma$. (**A**). Model V is similar to the previous one, but the gene expression process is split into the transcription and translation steps as follows from Eq. (12) (**B**). Model III considers that the number of TFs regulating CRS is lagged due to the finite time consumed during processes such as translation, elongation, or translocation (**C**). In all three cases, the architecture of CRS is the same.

This model is schematized in Fig. 4B. As shown in Appendix B of Supplementary Material, this model is associated with a five-order characteristic polynomial. An analytical study of this case for the entire parameter space is infeasible; however, we have verified its stability over a large region of the parameter space (see Supplementary Material). The number of equations in model V can also be reduced by introducing delay variables with the linear chain trick. To use the linear chain trick, we introduce the variable change, $m'=\alpha /\gamma \ m$. Therefore, we can rewrite the last two equations in (12) as

$$\begin{aligned} \dot{m'}= & {} \frac{\alpha _m \alpha }{\gamma } a_0 - \gamma _m m' \\ \dot{c}= & {} \gamma m' - \gamma c \end{aligned}$$

Following^25^, we have $c\left( t\right) = \int _{-\infty }^t \gamma e^{-\gamma (t-s)} m'\left( s\right) ds$, by changing variable $(t-s) \longrightarrow \tau$ we obtain

13 $$\begin{aligned} c\left( t\right) =\int _0^{\infty } \gamma e^{-\gamma \tau } m'\left( t-\tau \right) d\tau =\int _0^{\infty } K_{\gamma }(\tau ) m'\left( t-\tau \right) d\tau =D_{\gamma }\left[ m' \right] \end{aligned}$$

where $D_{\gamma }\left[ m' \right]$ is the normalized delay operator acting over $m'$ and $K_{\gamma }(\tau )$ is the Gamma distributed delay kernel of order 1, also known as weak delay kernel. Therefore, we can rewrite Eq. (12) as

14 $$\begin{aligned} \dot{a_0}= & {} - k_{01} a_0 D_{\gamma }\left[ m' \right] + k_{10} a_1 \nonumber \\ \dot{a_1}= & {} - k_{12} a_1 D_{\gamma }\left[ m' \right] + k_{21} a_2 + k_{01} a_0 D_{\gamma }\left[ m' \right] - k_{10} a_1 \nonumber \\ \dot{a_2}= & {} - k_{23} a_2 D_{\gamma }\left[ m' \right] + k_{32} (1 - a_0 - a_1 - a_2) + k_{12} a_1 D_{\gamma }\left[ m' \right] - k_{21} a_2 \nonumber \\ \dot{m'}= & {} \frac{\alpha _m \alpha }{\gamma } a_0 - \gamma _m m'. \end{aligned}$$

These equations resemble model IV but with a distributed delay kernel, as schematized in Fig. 4C. The model in Eq. (14) is also asymptotically stable, and its associated characteristic polynomial has the same order as the model V (see Appendix C of Supplementary Material). This is expected because the result of the linear chain trick can be understood as a reduction to integro-differential equations rather than an approximation.

**Figure 5:**
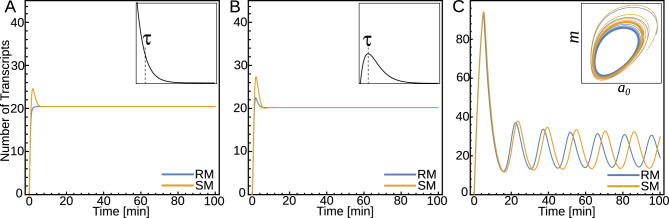
Temporal evolution of the single-gene circuit model corresponding to Eq. (14) with weak delay kernel for recruitment (blue line) and stabilization (magenta line) cooperative binding mechanisms, the weak distributed kernel function is depicted in the inset (**A**). Temporal evolution of Eq. (14), but with a strong delay kernel, the strong distributed kernel function is depicted in the inset (**B**). Temporal evolution of Eq. (14), but with a discrete delay, the inset depicts the trajectories in the phase plane (**C**). Parameter values: $p=0.246$, $q=30$, $\epsilon =10$, $\alpha _m=33$, $\gamma _m=0.23$, $\alpha =4.5$, $\gamma =4.6$.

A delay kernel is a weighting function that, in this case, indicates how much emphasis should be given to the protein concentration at earlier times to determine the present effect on CRS. In the case above, the weak delay kernel is a direct consequence of assuming that translation is a process occurring at a given rate. Nevertheless, as we saw in the previous section, the order of the delay kernel is associated with the number of processes replaced. To illustrate the effect of the inclusion of further processes in the model, we will include unspecific processes by considering a Gamma-distributed delay kernel of order 2 (known as the strong delay kernel) and also a model with an infinite-order kernel (discrete delay). In the strong delay case, $n=2$, we have a sixth-order characteristic polynomial, while in the case of discrete delay, the characteristic equation becomes a transcendental equation. As shown in Fig. 5A,B the steady state is the same in the three cases, as expected; however, systems with different cooperative binding mechanisms display different transients. Further, the fix-point can lose its stability depending on the order of the delay kernel. In particular, Fig. 5C shows that the model with a discrete kernel presents sustained oscillations and that the frequency of these oscillations depends on the cooperative binding mechanism that is acting. The different behavior between the cooperative mechanisms is bypassed when using the Hill function as a phenomenological regulatory function. In addition, the use of a phenomenological regulatory function (i.e., an instantaneous regulatory response) also neglects the interplay among the characteristic times involved in synthesis/degradation processes and the dynamics of the CRS.

We also explore how the CRS dynamics affect the instability of the single-gene circuit governed by Eq. (14) with discrete delay by varying the kinetic rates *p* and *q*, but keeping $K_d$ and the rates that control the synthesis and degradation processes fixed. This is possible because the resulting Adair regulatory function depends only on the kinetic rates through the quotient $k_{ij}/k_{ji}$. To this purpose, the parameter values for these processes are similar to Fig. 2: $\alpha _m=33$ molec/min, $\gamma _m=0.23$ molec/min, $\alpha =4.5$ molec/(molec.min), $\tau = 3.5$ min. While the parameters associated with CRS dynamics are set to $\epsilon =8.5$, $p = f\times 0.1$ min^-1^ and $q = f\times 43$ min^-1^ where *f* is a variable factor that decreases (or increases) the kinetic rates without affecting the regulatory function. With the values above for CRS dynamics, the parameters associated with the regulatory function yield $K_d = 40.1$ molec and $n_H = 2.21$ (yellow curve in Fig. 3), while the FT residence time ($q^{-1}$) ranges between 15.5 and 46.5 s.

**Figure 6:**
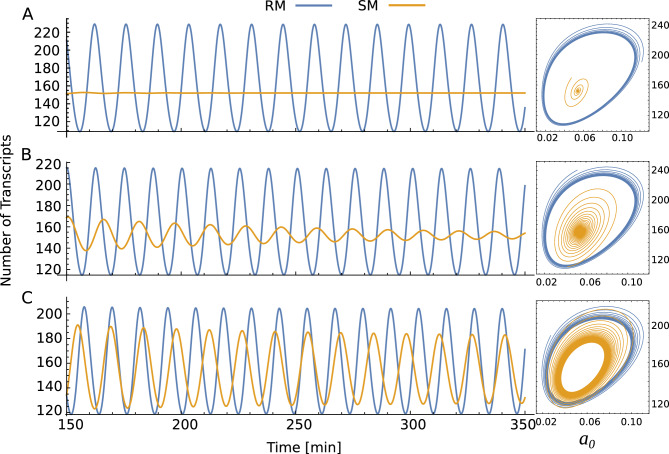
The effects of CRS dynamics on system stability. Time course and trajectories in the phase plane of a single-gene circuit model corresponding to Eq. (14) with a discrete delay kernel with different kinetic-factor values: $f=0.03$ (**A**), $f=0.06$ (**B**), and $f=0.09$ (**C**). The simulations are both for the recruitment (RM blue line) and stabilization (SM yellow line) cooperative binding mechanisms.

Figure 6 illustrates the effect of the CRS dynamics on the instability of the transcriptional oscillator with discrete delay. As regulatory function and steady-state depend on the ratio *p*/*q*, the parameter *f* only alters the relations between the dynamics of CRS and the rates of synthesis and degradation processes. We found that at slow CRS kinetics, the stability of the system depends on which cooperative binding mechanism is acting. Thus, Fig. 6A,B shows that while the system with SM presents a stable spiral behavior (yellow trajectory), the RM is associated with sustainable oscillations (blue trajectory). By increasing the binding and unbinding rates *p* and *q* through increasing factor *f*, we observe that systems with SM can also become unstable. We also observe that the frequency of the oscillations increases with *f*. On the other hand, Fig. 6C shows that when both systems reach the regime of sustainable oscillation, there is an evident difference in the frequencies of the oscillations associated with each cooperative binding mechanism. It is worth noting that the mechanism RM is the one associated with the fastest oscillations. These examples show that the fix-point can lose its stability depending on the details of the cooperative binding mechanism. Further, the amplitude and frequency of the oscillations also depend on the cooperative binding mechanism and kinetic rates of CRS. Consequently, important features of observed phenotypes can be misinterpreted when using the instantaneous regulatory function approximation. The results obtained for the model operating under the SM are consistent with the requirements for the occurrence of oscillations observed for Hes7 variants of different half-lives^29^. However, this is not the case for the RM, where sustained oscillations are maintained in the range of parameter values studied. This result suggests that cooperativity operating in the CRS of these genes would be of the SM type.

**Figure 7:**
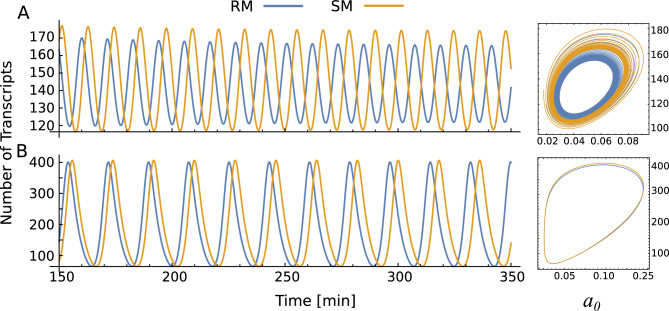
Time course and trajectories in the phase plane of single-gene circuit model corresponding to Eq. (14) with a discrete delay kernel with different kinetic-factor values: $f=1.5$ and $\tau =3.5$ (**A**), same *f* but $\tau =5.5$ (**B**). The simulations are both for the recruitment (blue line) and stabilization (yellow line) cooperative binding mechanisms.

Another important question to address is about the validity of the instantaneous approximation. It is expected that instantaneous approximation works fine when the synthesis/degradation processes (or other parameters related to the delay variables) are slow in comparison with CRS dynamics. Figure 7 shows that the observed difference among cooperative mechanisms decreases when the rates associated with the CRS dynamics increase regarding the rates of synthesis and degradation processes (Fig. 7A). Further, as expected, when the parameter associated with lag increases in systems with discrete delay, we also observe that the difference in the frequencies of the oscillations associated with each cooperative binding mechanism vanishes (Fig. 7B).

## Discussion and conclusion

Systems biology focuses on understanding the emergent properties of biological networks through mathematical modeling. In the case of a single-gene oscillatory circuit, most models are based on a phenomenological regulatory function, characterized by the dissociation constant $K_d$ and Hill exponent $n_H$, which summarize all interactions among TFs and CRS. These simplifications can be useful for exploring complex circuit topologies; however, even in the realm of low-dimension models, like the somite segmentation clock^30–32^, the information about the interaction between elements within these networks, as included in the models, is reduced to minimal. Consequently, simple models might overlook important aspects related to the intrinsic dynamics of the CRS. In this context, we have presented a single-gene circuit that represses its own transcription, and we show that, according to the levels of detail incorporated, it exhibits different behaviors. For instance, Model I, which represents the synthesis and degradation processes instantaneously, yields a stable fixed-point solution. However, Model II, which splits the expression process into transcription and translation, presents a stable spiral. Further, Model III exhibits sustained oscillations. This example concludes that oversimplifying multi-step processes through instantaneous representations can yield misleading outcomes. Thus, for a more precise characterization of gene circuits, the underlying interactions between elements must be quantitatively characterized. While estimating parameters $K_d$ and $n_H$ from dose-response curves is relatively straightforward, accurately measuring binding parameters presents a more complex challenge. Recently, new techniques to determine binding parameters have been developed. These studies have revealed that both binding and unbinding rates can vary significantly, by several orders of magnitude^33,34^. In particular, single-molecule tracking approaches report that TFs have average residence times at specific regulatory sites on the order of 2–100 s^34^, while for transient interactions with non-specific DNA binding sites is less than 1 s^35^. These measurements indicate a wide range of variation for parameter *q*, leading to a door open to precise discussion about the results obtained with the instantaneous approach of the CRS in the context of transcriptional oscillation modeling.

A critical aspect of transcriptional oscillator models is the relationship between the half-lives of mRNA and proteins and the kinetics associated with the processes of TF binding/unbinding to DNA^36^. For example, the model of Lewis^15^ generates oscillations when the lifetimes of the mRNA and protein are very short compared with the rate constants for RNA and protein synthesis^37^. While the role of the degradation rate in these oscillations is beginning to be elucidated^29^, less is known about the role of the multiple binding sites regulating her/hes genes. Although theoretical studies with multiple regulatory sites show a decreased oscillatory frequency^38^. This effect could be a consequence of a combination of greater ultrasensitivity for the repression of the CRS and a greater effective delay in the explicit dynamics of the CRS. In this context, in “Single-gene oscillator models with explicit CRS dynamics”, we have studied the behavior of the system governed by Eq. (14) with discrete delay by varying the kinetic rates *p* and *q* but keeping $K_d$ fixed. Our detailed model shows that, at slow CRS kinetics, the presence or not of oscillations depends on the assumed cooperativity mechanism, an aspect that would be ignored with a phenomenological simplification. Furthermore, in the oscillatory regime, the frequency and amplitude achieved depend on the proposed mechanism. Finally, it is not surprising that instantaneous approximation works when parameters related to lagged variables are high (slow processes) concerning CRS dynamics. However, the analysis of our detailed model with a discrete delay shows that the frequencies of oscillations can depend on the binding mechanism considered when the time lag is small.

In summary, our analysis of an autoinhibitory single-gene circuit by models with different detail levels shows that a model built under the same hypotheses, but with different levels of detail considered, leads to different results. On the one hand, describing the elongation processes as step-to-step processes can introduce instabilities and oscillations not seen in an instantaneous simplification. Furthermore, incorporating a detailed description of the CRS dynamics, usually modeled by a phenomenological regulatory function, can lead to instability, depending on the cooperative binding mechanism that is acting.

### Supplementary Information

Supplementary Information.

## Data Availability

All data generated or analysed during this study are included in this published article and its supplementary information files. Python notebooks to study our models are available on GitHub (https://github.com/ldiambra/lessonsfromsinglegene).
