# Supplementary material for: Simplifications and approximations in a single-gene circuit modeling
Source: Sci Rep. 2024 May 31;14:12498. doi: 10.1038/s41598-024-63265-8 (PMC11143231; doi:10.1038/s41598-024-63265-8)
Supplement: Supplementary file 1 — Supplementary Information. [file 41598_2024_63265_MOESM1_ESM.pdf]

Supplementary Material
Simplifications and approximations in a single-gene circuit modeling

Alejandro Barton[1,2], Pablo Sesin[3] and Luis Diambra[1,2,*]

[1] Centro Regional de Estudios Genómicos, Universidad Nacional de La Plata, Argentina.
[2] Consejo Nacional de Investigaciones Científicas y Técnicas, Argentina
[3] Departamento de Física Teórica, Comisión Nacional de Energía Atómica, Argentina.
[*] Corresponding author: ldiambra@gmail.com

Appendix A

The steady state of model IV is defined when the variation of all chemical species is zero. Specifically, this occurs when we solve the following system of equations, which represent the equilibrium conditions of our biochemical network. Thus, we can write

$$\begin{aligned} &a_0 = \gamma/\alpha\ c \\ &a_1 = \gamma/\alpha\ K_1 c^2 \\ &a_2 = \gamma/\alpha\ K_1 K_2 c^3 \\ &\alpha - \gamma c - \gamma K_1 c^2 - \gamma K_1 K_2 c^3 - \gamma K_1 K_2 K_3 c^4 = 0. \end{aligned} \tag{1}$$

Each variable a_0, a_1, a_2, and c represents a specific chemical species in our model, where c might be the concentration of a key regulatory protein. The last equation in (**??**) is a polynomial with four roots that define the fixed points of the system. These roots are two complex conjugated, one real negative and one real positive. This last fixed point has an analytic expression and it is the steady state of interest in our problem and will be denoted by $\mathbf{S} = (a_0^*, a_1^* a_2^*, c^*)$. To study the stability of this state we linearize the system about the steady-state $\mathbf{S}$ and compute the Jacobian

matrix evaluated in **S**:

$$\begin{pmatrix} -k_{01}c^* & k_{10} & 0 & -k_{01}a_0^* \\ k_{01}c^* & -(k_{10}+k_{12}c^*) & k_{21} & (k_{01}a_0^* - k_{12}a_1^*) \\ -k_{32} & k_{12}c^* - k_{32} & -(k_{21}+k_{23}c^*+k_{32}) & (k_{12}a_1^* - k_{23}a_2^*) \\ \alpha & 0 & 0 & -\gamma \end{pmatrix}$$

The associated eigenvalues λ_i are given by the roots of the polynomial: $b_0\lambda^4 + b_1\lambda^3 + b_2\lambda^2 + b_3\lambda + b_4$. Taking into account the relationships between a_i and c at the steady state in (**??**), the coefficients b_i can be written as:

$$\begin{aligned}
b_0 &= 1,\\
b_1 &= c^*(k_{01}+k_{12}+k_{23}) + \gamma + k_{10} + k_{21} + k_{32},\\
b_2 &= c^{*2}(k_{01}k_{12} + k_{01}k_{23} + k_{12}k_{23}) + c^*(k_{01}k_{21} + k_{01}k_{32} + k_{10}k_{23} + k_{12}k_{32})\\
&\quad + c^*\gamma(2k_{01} + k_{12} + k_{23}) + \gamma(k_{10} + k_{21} + k_{32}) + k_{10}k_{21} + k_{10}k_{32} + k_{21}k_{32},\\
b_3 &= c^{*2}\gamma(3k_{01}k_{12} + 2k_{01}k_{23} + k_{12}k_{23}) + c^*\gamma(2k_{01}k_{21} + 2k_{01}k_{32} + k_{10}k_{23} + k_{12}k_{32})\\
&\quad + \gamma(k_{10}k_{21} + c^{*3}k_{01}k_{12}k_{23} + c^{*2}k_{01}k_{12}k_{32} + c^*k_{01}k_{21}k_{32} + k_{10}k_{32} + k_{21}k_{32})\\
&\quad + k_{10}k_{21}k_{32},\\
b_4 &= \gamma(4c^{*3}k_{01}k_{12}k_{23} + 3c^{*2}k_{01}k_{12}k_{32} + 2c^*k_{01}k_{21}k_{32} + k_{10}k_{21}k_{32},
\end{aligned}$$

Note that the necessary condition for the stability is satisfied if all the coefficients $b_i > 0$, which is granted because they are sums of positive terms. Of course, we are considering all parameters to be positive. Following the Routh-Hurwitz stability criterion: The roots of the characteristic polynomial have negative real parts if and only if all the principal diagonal minors, Δ_i, of the Hurwitz matrix are positive. That is:

$$b_0 > 0,\ \ \Delta_1 = b_1 > 0,\ \ \Delta_2 = \begin{vmatrix} b_1 & b_0 \\ b_3 & b_2 \end{vmatrix} = b_1b_2 - b_0b_3 > 0,$$

$$\Delta_3 = \begin{vmatrix} b_1 & b_0 & 0 \\ b_3 & b_2 & b_1 \\ 0 & b_4 & b_3 \end{vmatrix} = b_1b_2b_3 - b_1^2b_4 - b_0b_3^2 > 0,\ \ \Delta_4 = b_4\Delta_3 > 0$$

It is possible to demonstrate that principal diagonal minors Δ_2 and Δ_3 are summation positive terms. As all b_i are positive we have that Δ_1 and Δ_4 are also positive we can conclude that the real positive solution of (**??**) is asymptotic stable for all parameters values independent on which cooperative binding mechanism is acting. However, when considering time-delay variables, we show that the stability of the fixed point can depend on the cooperative mechanism.

Appendix B

For the model V, the steady state of protein concentration, denoted by p^*, is determined by following relationship

$$-\frac{\alpha_m \alpha}{\gamma_m \gamma} + c^* + K_1 c^{*2} + K_1 K_2 c^{*3} + K_1 K_2 K_3 c^{*4} = 0, \tag{2}$$

where $K_1 = k_{01}/k_{10}$, $K_2 = k_{12}/k_{21}$ and $K_3 = k_{23}/k_{32}$ are the equilibrium constants. The steady-state associated with the other variables can be written in terms of c^* as follow:

$$\begin{aligned} m^* &= \frac{\gamma}{\alpha}\, c^* & a_0^* &= \frac{\gamma_m \gamma}{\alpha_m \alpha}\, c^* \\ a_1^* &= \frac{\gamma_m \gamma}{\alpha_m \alpha}\, K_1 c^{*2} & a_2^* &= \frac{\gamma_m \gamma}{\alpha_m \alpha}\, K_1 K_2 c^{*3} \end{aligned} \tag{3}$$

The equation (**??**) is a polynomial have four roots. The steady state of interest in our problem is in two branches depending on ϵ value, it has a complicated form but closed expression:

$$\begin{aligned} c^*(g,x,\epsilon)^{\pm} =& \frac{1}{4\epsilon^2 x}\left(\sqrt{2(9-8\epsilon) - 4\epsilon F(g,x,\epsilon) \pm \frac{2\,(4\epsilon^2+6\epsilon-9)\,(3-2\epsilon)}{\sqrt{4\epsilon F(g,x,\epsilon)+(9-8\epsilon)}}}\right. \\ & \left. - \sqrt{4\epsilon F(g,x,\epsilon)+(9-8\epsilon)} - 3\right), \end{aligned}$$

where $x = p/q$, and $g = \frac{\gamma_m \gamma}{\alpha_m \alpha}$. $\pm$ indicates the correct branch for a real positive solution ($+$ when $1 < \epsilon < 3/2$, and $-$ when $3/2 < \epsilon$). The function F is given by:

$$F(g,x,\epsilon) = \frac{3(1-\epsilon)g - 4\epsilon^3 x}{3g\sqrt[3]{\frac{f_1(g,x,\epsilon)+f_2(g,x,\epsilon)}{2g}}} + \sqrt[3]{\frac{f_1(g,x,\epsilon)+f_2(g,x,\epsilon)}{2g}},$$

and

$$\begin{aligned} f_1(g,x,\epsilon) &= \left(\epsilon^3 - 3\epsilon + 2\right) g - \epsilon^2(9-8\epsilon)x, \\ f_2(g,x,\epsilon) &= \sqrt{f_1(g,x,\epsilon)^2 - \frac{4\left(3(1-\epsilon)g - 4\epsilon^3 x\right)^3}{27g}}, \end{aligned}$$

To study the stability of this state we linearize the system about the

steady-state **S** and compute the Jacobian matrix evaluated in **S**:

$$\begin{pmatrix} -k_{01}c^* & k_{10} & 0 & 0 & -k_{01}a_0^* \\ k_{01}c^* & -(k_{10}+k_{12}c^*) & k_{21} & 0 & (k_{01}a_0^* - k_{12}a_1^*) \\ -k_{32} & k_{12}c^* - k_{32} & -(k_{21}+k_{23}c^*+k_{32}) & 0 & (k_{12}a_1^* - k_{23}a_2^*) \\ \alpha_m & 0 & 0 & -\gamma_m & 0 \\ 0 & 0 & 0 & \alpha & -\gamma \end{pmatrix}$$

This matrix leads us to a characteristic polynomial with order five: $b_0\lambda^5 + b_1\lambda^4 + b_2\lambda^3 + b_3\lambda^2 + b_2\lambda + b_5$, where the coefficients b_i are given by:

$$\begin{aligned} b_0 &= 1, \\ b_1 &= c^*(k_{01}+k_{12}+k_{23}) + k_{10} + k_{21} + k_{32} + (\gamma_m+\gamma), \\ b_2 &= c^{*2}(k_{01}(k_{12}+k_{23}) + k_{12}k_{23}) + c^*(\gamma_m(k_{01}+k_{12}+k_{23}) + k_{01}(k_{21}+k_{32}+\gamma) \\ &\quad +k_{23}(k_{10}+\gamma_p) + k_{12}(k_{32}+\gamma)) + (\gamma_m+\gamma)(k_{10}+k_{21}+k_{32}) + (\gamma_m\gamma) \\ &\quad +k_{10}k_{21} + k_{10}k_{32} + k_{21}k_{32}, \\ b_3 &= c^{*3}k_{01}k_{12}k_{23} + c^{*2}((\gamma_m+\gamma)(k_{01}k_{23}+k_{12}k_{23}+k_{01}k_{12}) + k_{01}k_{12}k_{32}) \\ &\quad +c^*((\gamma_m+\gamma)(k_{01}k_{21}+k_{01}k_{32}+k_{10}k_{23}+k_{12}k_{32}) + \gamma_m\gamma(2k_{01}+k_{12}+k_{23}) + k_{01}k_{21}k_{32}) \\ &\quad +(\gamma_m+\gamma)(k_{10}k_{21}+k_{10}k_{32}+k_{21}k_{32}) + \gamma_m\gamma(k_{10}+k_{21}+k_{32}) + k_{10}k_{21}k_{32}, \\ b_4 &= c^{*3}k_{01}k_{12}k_{23}(\gamma_m+\gamma) + c^{*2}(k_{01}k_{12}k_{32}(\gamma_m+\gamma) + \gamma_m\gamma(3k_{01}k_{12}+2k_{01}k_{23}+k_{12}k_{23})) \\ &\quad +c^*(\gamma_m\gamma(2k_{01}(k_{21}+k_{32}) + k_{10}k_{23} + k_{12}k_{32}) + k_{01}k_{21}k_{32}(\gamma_m+\gamma)) \\ &\quad +k_{10}k_{21}k_{32}(\gamma_m+\gamma) + \gamma_m\gamma(k_{10}k_{21}+k_{10}k_{32}+k_{21}k_{32}), \\ b_5 &= \gamma_m\gamma\left(4c^{*3}k_{01}k_{12}k_{23} + 3c^{*2}k_{01}k_{12}k_{32} + 2c^*k_{01}k_{21}k_{32} + k_{10}k_{21}k_{32}\right) \end{aligned} \quad (4)$$

Note that the necessary condition for the stability is satisfied if all the coefficients $b_i > 0$, which is granted because they are sums of positive terms. Following the Routh-Hurwitz stability criterion: The roots of the characteristic polynomial have negative real parts if and only if all the principal diagonal minors, Δ_i, of the Hurwitz matrix are positive. That is:

$$b_0 > 0, \;\; \Delta_1 = b_1 > 0, \;\; \Delta_2 = \begin{vmatrix} b_1 & b_0 \\ b_3 & b_2 \end{vmatrix} = b_1b_2 - b_0b_3 > 0,$$

$$\Delta_3 = \begin{vmatrix} b_1 & b_0 & 0 \\ b_3 & b_2 & b_1 \\ b_5 & b_4 & b_3 \end{vmatrix} = b_1b_2b_3 - b_1^2b_4 - b_0b_3^2 + b_0b_1b_5 > 0,$$

$$\Delta_4 = \begin{vmatrix} b_1 & b_0 & 0 & 0 \\ b_3 & b_2 & b_1 & b_0 \\ b_5 & b_4 & b_3 & b_2 \\ 0 & 0 & b_5 & b_4 \end{vmatrix}, \;\; \Delta_5 = b_5\Delta_4 > 0$$

Taking into account the relationships between a_i and c in Eq. (**??**) at the steady state, we can show that the principal diagonal minors Δ_1, Δ_2 and Δ_3 are both summation of positive terms. We cannot determine the sign of Δ_4 in an analytical fashion for the whole parameter space. However, we have found numerically that it is positive in a large region of the parameter space. As all b_i are positive we have that Δ_1 and Δ_5 are also positive and can conclude that the real positive solution of (**??**) is asymptotic stable for a large region of parameters values.

Appendix C

In this section, we delve deeper into the dynamics of the model governed by Eqs. (14) by performing a linear stability analysis, considering a more general case with a Gamma distributed delay kernel of order n. This approach allows us to account for temporal delays in biological responses, represented by the delay operator D^n_τ. Of course for $n = 1$ we have $D^1_\tau = D_\gamma$. Before performing this analysis it is important to note that fixed points of the system without delay are also fixed points of the delayed system. However, delays can change the stability of fixed points, even without altering their position with respect to the system without delay.

In the present case, the model without delay corresponds to model IV studied in Appendix A, but where variable c changes to m'. For the sake of simplifying the notation, we will remove the tilde from m'. The steady state, now denoted by $(a^*_0, a^*_1 a^*_2, m^*)$, is determined for a set of equations formally identical to (**??**). Linearizing around the fix point $\mathbf{S} = (a^*_0, a^*_1 a^*_2, m^*)$, with respect to the variables: a_0, a_1, a_2, m and $m(t-\tau)$ and obtain linear equations for small deviations $\overline{a}_i = a_i - a^*_i$ and $\overline{m} = m - m^*$

$$
\begin{aligned}
\dot{\overline{a}}_0 &= -k_{01} m^* \overline{a}_0 + k_{10}\overline{a}_1 - k_{01} a^*_0 D^n_\tau[\overline{m}] \\
\dot{\overline{a}}_1 &= -k_{12}\overline{a}_1 m^* + k_{21}\overline{a}_2 + k_{01} m^* \overline{a}_0 - k_{10}\overline{a}_1 \\
&\quad -k_{12} a^*_1 D^n_\tau[\overline{m}] + k_{01} a^*_0 D^n_\tau[\overline{m}] \\
\dot{\overline{a}}_2 &= -k_{23}\overline{a}_2 m^* - k_{32}(\overline{a}_0 + \overline{a}_1 + \overline{a}_2) + k_{12} m^* \overline{a}_1 - k_{21}\overline{a}_2 \\
&\quad -k_{23} a^*_2 D^n_\tau[\overline{m}] + k_{12} a^*_1 D^n_\tau[\overline{m}] \\
\dot{\overline{m}} &= \alpha_m \overline{a}_0 - \gamma_m \overline{m}
\end{aligned}
\qquad (5)
$$

As (**??**) is a linear system, we propose a solution in the form $A_i \exp(wt)$ for variables $\overline{a}_i$ and similar one $C\exp(wt)$ for $\overline{m}$, where the amplitude of deviation A_i, C and w are constants to be determined. Thus, replacing this

solution in (**??**) and taking into account that

$$D_\tau^n\left[\overline{m}(t)\right] = \int_0^\infty K_n(\tau) C \exp w(t-\tau)\, d\tau = C \exp(wt) \frac{\tau^n}{(\tau+w)^n},$$

This calculation holds for complex w satisfying $\mathfrak{Re}(w) > -\tau$. One can arrive at the secular equation $(J - w\mathbb{I})\mathbf{A} = 0$, where $\mathbb{I}$ is the identity matrix and $\mathbf{A} = (A_0, A_1, A_2, C)$. J is the Jacobian matrix of (**??**) evaluated in the steady state $\mathbf{S}$ which is given by:

$$\begin{pmatrix} -k_{01}m^* & k_{10} & 0 & -k_{01}a_0^* \frac{\tau^n}{(\tau+w)^n} \\ k_{01}m^* & -(k_{10}+k_{12}m^*) & k_{21} & (k_{01}a_0^* - k_{12}a_1^*)\frac{\tau^n}{(\tau+w)^n} \\ -k_{32} & k_{12}m^* - k_{32} & -(k_{21}+k_{23}m^*+k_{32}) & (k_{12}a_1^* - k_{23}a_2^*)\frac{\tau^n}{(\tau+w)^n} \\ \alpha_m & 0 & 0 & -\gamma_m \end{pmatrix}$$

The amplitude of perturbation $\mathbf{A}$ can be different from zero, if and only if $\det(J - w\mathbb{I}) = 0$. This condition leads us to a characteristic polynomial with order $4+n$. In the weak delay kernel case, $n = 1$, we have: $b_0\lambda^5 + b_1\lambda^4 + b_2\lambda^3 + b_3\lambda^2 + b_2\lambda + b_5$, where the coefficients b_i are formally the same that (**??**), which implies that the steady state is asymptotically stable. Due to the complexity of the problem, we do not compute the stability for the strong delay case, $n = 2$.
